# The Imperial Vaccination League

**Published:** 1902-08-30

**Authors:** 


### The Hospital

A Journal of

The Medical Sciences and Hospital Administration.

Vol. XXXII.—No. 831. Edited by Sir Henry Burdett, K.C.B., and Solomon C. Smith, M.D., M.R.C.P. [August 30, 1902.

### THE IMPERIAL VACCINATION LEAGUE.

The formation of the Imperial Vaccination League, for the specific purposes of instructing the public with regard to the advantages offered by vaccination, and of instructing the Legislature with regard to the enactments by which the largest degree of security can be obtained, is an event of considerable significance, which may not impossibly be productive of far-reaching effects. The controversy as to vaccination has hitherto been conducted, in this country, under singularly unfair conditions; on the one side by a skilfully-arranged organisation, working through the agency of well-selected and highly-paid writers and lecturers; on the other side by an irregular force of amateur skirmishers, many of whom have been very imperfectly acquainted with the subject concerning which they have endeavoured to diffuse enlightenment, while others have been singularly wanting in the power of expressing their knowledge in a lucid and convincing manner. The general result has been that, while the advocates of vaccination have had the best of the facts, they have by no means had the best of the arguments, and on more than one occasion they have walked straight into statistical and other traps prepared for them by the astuteness of their adversaries. It is devoutly to be hoped, therefore, that irresponsible amateurs will now retire from a field in which they have not succeeded in winning laurels; that "The Mother of a Family" will cease from troubling; and that "A Constant Reader" will be at rest. It is equally to be hoped that the executive of the new organisation will walk warily, will exercise discretion in the choice of those who are to be empowered to speak and write under its authority, and, alike in its statements and in its recommendations, will strive to lead, and not to drive, the various elements which unite to make up public opinion. The reference to Germany in the first manifesto of the League is perhaps hardly judicious; because, although many people will agree with the wish that this country were as well protected as Germany, comparatively few might be content that this protection should be afforded by German methods. Such methods would at least admit of being represented as antagonistic to, if not as destructive of, English liberties; and we may so far parody the words of a late archbishop as to say, with his important, but frequently omitted, reservation, "if compelled to make the choice," that many people might be found to declare that they would rather see Englishmen free than protected. If the League does its work well, the truth about vaccination will at no distant time be established beyond the reach of further controversy; and, whenever this time comes, the enforcement of the practice will only be needed in the case of ignorant or neglectful parents, or possibly in the slumberous recesses of the Local Government Board, among politicians who live in the practice of not governing. That vaccination should be required by law, and that the law should make no provision for securing an adequate supply of trustworthy lymph, is one of the things which no fellow can be expected to understand. As the *Times* said, when attention was first called to the facts, they are suggestive of comic opera rather than of civilised administration.

It is not only in respect of vaccination that we recognise in the establishment of the League an event which is full of promise for the future. It constitutes the first step ever taken in this country for the systematic instruction of the public upon a medical question; and, if it should prove to be successful, it can hardly fail to bring home to the corporate mind of the medical profession the necessity of "educating its masters." Medical men have lately complained that medicine is not represented in Parliament, but they do not, as far as we have seen, indicate any manner in which the desired "representation" could be secured. Fancy franchises have never found favour with Englishmen, and there is practically no chance whatever that the Reform Bill of the future will contain any clause giving direct representation to doctors. If to doctors, why not to electrical engineers, to stockbrokers, or to architects? The truth is, that the somewhat scant consideration often shown to the medical profession by the legislature has been a result, not of any disposition to deal hardly with a class, but of want of knowledge of the importance of the issues involved. The legislative requirement of certificates of the causes of death, to be given without payment, was due to pure ignorance of the character of the demand. The average member of Parliament thinks that fatal diseases are easily recognisable entities, as Miss Nightingale said, "like dogs and cats"; and, believing that every doctor would know at a glance what his patient died of, would think it no hardship to call upon the aforesaid doctor to write and sign the statement. The knowledge that the call was really for the performance of a difficult and highly responsible duty, of great importance to the public, would speedily be productive of reform; and what is true of certification is at least equally true of the circumstances which underlie other pet grievances of medical agitators.

